# A Network-based Approach for Predicting Missing Pathway Interactions

**DOI:** 10.1371/journal.pcbi.1002640

**Published:** 2012-08-16

**Authors:** Saket Navlakha, Anthony Gitter, Ziv Bar-Joseph

**Affiliations:** School of Computer Science and Lane Center for Computational Biology, Carnegie Mellon University, Pittsburgh, Pennsylvania, United States of America; The Pennsylvania State University, United States of America

## Abstract

Embedded within large-scale protein interaction networks are signaling pathways that encode response cascades in the cell. Unfortunately, even for well-studied species like *S. cerevisiae*, only a fraction of all true protein interactions are known, which makes it difficult to reason about the exact flow of signals and the corresponding causal relations in the network. To help address this problem, we introduce a framework for predicting new interactions that aid connectivity between upstream proteins (sources) and downstream transcription factors (targets) of a particular pathway. Our algorithms attempt to globally minimize the distance between sources and targets by finding a small set of shortcut edges to add to the network. Unlike existing algorithms for predicting general protein interactions, by focusing on proteins involved in specific responses our approach homes-in on pathway-consistent interactions. We applied our method to extend pathways in osmotic stress response in yeast and identified several missing interactions, some of which are supported by published reports. We also performed experiments that support a novel interaction not previously reported. Our framework is general and may be applicable to edge prediction problems in other domains.

## Author Summary

Networks of protein interactions encode a variety of molecular processes occurring in the cell. Embedded within these networks are important subnetworks called signaling pathways. Pathways are initiated by upstream proteins (called sources) that receive signals from the environment and trigger a cascade of information to downstream proteins (targets). Modeling the interactions that occur within this cascade is important because pathway disruption has been linked to several diseases. Further, the interactions help us better understand how cells respond to various conditions and environments. Unfortunately, interaction networks today are largely incomplete, which makes this analysis difficult. We provide a framework to model missing interactions in pathways by searching for interactions that putatively result in quicker and more efficient source-target cascades. We find that we can substantially shorten source-target distances with only a few additional edges and that many of our predicted edges have support in several knowledge databases and literature reports. We believe our approach will be useful to identify interesting and important pathway-centric interactions that have been missed by previous experimental assays.

## Introduction

Networks of protein interactions can reveal how complex molecular processes are activated in the cell. However, even for model species, only a fraction of true physical interactions are known [1], [2] and experimental verification of all remaining potential interactions is unlikely in the near future. Furthermore, interactions are often condition- or tissue-specific [3] while current experimental methods often focus on one condition and one cell type [4]. Thus, computational techniques to predict protein interactions have flourished as a means to build more complete interaction maps [5], [6].

Signaling pathways are subnetworks of proteins that communicate via a series of interactions and are often only activated under specific conditions (e.g. stress response, development, etc.). Perturbations of proteins within such pathways have been linked to several diseases [7]. In addition, pathways are often conserved, thus studying their interactions in model organisms may help elucidate cellular response mechanisms in other organisms [8].

Signaling pathways typically contain upstream proteins (e.g. receptors on the cell's surface) that sense changes in the environment or that are directly involved in host-pathogen interactions. These proteins trigger a signaling cascade that leads to downstream transcription factors (TFs), which consequently carry forth regulatory programs. The former set of proteins can be considered *sources* that transmit information to a set of *targets*. Experimental protocols can infer source proteins based on their interactions with external stimuli (e.g. host-pathogen interactions [9]), and likewise targets can be determined via expression or knockdown assays. This motivated several techniques that have been proposed to extract pathways from global interaction networks by searching for efficient and robust paths between the given sets of sources and targets [10]–[13]. These techniques, however, do not try to infer putative interactions that are *missing* from the network. We model this problem computationally by searching for missing edges that increase the network's ability to explain the signaling cascade from sources to targets.

Many methods have been proposed to computationally predict protein-protein interactions. These methods leverage a variety of data sources, including physical docking models and protein structure [14], [15], evidence based on orthologous proteins in related species [16], microarray expression profiles [17]–[21], literature mining [22], sequence-level features [23]–[27], or a combination of heterogeneous features to learn a predictive model or classifier [28]–[32] (for reviews, see [5], [6]). Network-only approaches range from completing defective cliques [33] to analyses based on the shared topology or the distance between two candidate proteins [34], [35] to embeddings of the network to find non-interacting but adjacent proteins in the new space [36], [37]. None of these approaches, however, leverage known sources and targets to make pathway-aware predictions. Further, most other approaches use local cues of similarity, whereas our approach attempts to optimize a global distance function. There has also been theoretical work on predicting “shortcut edges” in graphs to minimize the average shortest-path distance amongst all nodes in the graph [38] or the diameter of the graph [39]–[42]; however, these works also do not exploit specific sources and targets when making predictions.

**Figure 1:**
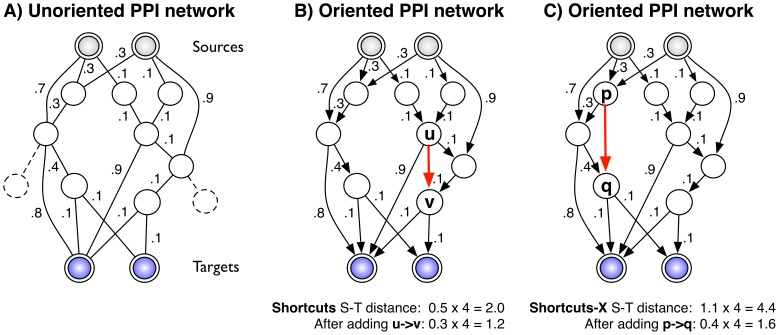
Overview of our approach. A) Example input network with sources, targets, and undirected edges. Each edge is given a weight (lower values indicate higher confidence). The total distance from each source to each target is 2.0. B) The corresponding oriented network. Nodes and edges that do not lie within a path of 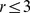 hops from any source-target pair are purged (shown dashed in A). The red arrow indicates an edge prediction (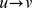) that globally minimizes the distance between each source and target using the Shortcuts objective function. The new distance is 1.2. C) The corresponding example using the Shortcuts-X objective function with 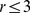. Here, the total hop-restricted distance between each source and target is higher (4.4) and the optimal edge, 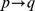 reduces the distance to 1.6.

In this paper, we propose a combinatorial optimization framework to identify missing interactions that putatively mediate the passage of signals within pathways. Formally, we seek the 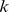 edges to add to the network that maximally decrease the shortest-path distances between sources and targets (Figure 1). We consider several variants of the problem: an unrestricted setting where long paths are allowed; a restricted setting where source-target paths are bounded by a maximum number of hops; and a setting where each target is only required to be regulated by a single source. In computational experiments using a confidence-weighted protein interaction network for *S. cerevisiae* under the high osmolarity glycerol (HOG) osmotic stress response pathway, we find that we can drastically reduce source-target distances via the addition of only a few edges. Several new interactions predicted by our method, while missing from current databases, are supported by the literature; other interactions are novel predictions. We selected one of our novel predictions, 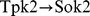, for condition-specific follow-up experiments. New knockout microarray experiments suggest that Sok2 is indeed functionally downstream of Tpk2 in the osmotic stress response, and previous evidence suggests that this could be due to Tpk2's direct phosphorylation of Sok2.

## Methods

We first present our framework for predicting missing edges in graphs based on their ability to connect a given set of sources and targets. We show that our collection of problems are NP-hard to solve optimally and describe two efficient greedy optimization algorithms to address them. We then describe our testing setup, followed by our computational and experimental results.

### A framework for pathway-consistent edge predictions

We assume we are given a directed protein interaction network 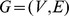 with nodes (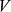) corresponding to proteins and edges (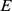) to physical interactions. Protein interaction networks inferred from high-throughput experiments are often noisy [2], [43], therefore we assume each edge is weighted by a value 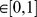 denoting our confidence in the interaction [13]. We also assume we are given a set of sources 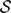 and targets 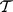. The sources are typically upstream proteins in pathways that initiate a signaling cascade to the downstream targets (transcription factors). Our goal is to predict missing (directed) edges that lie centrally “in-between” the sources and targets. These edges putatively belong to the pathway but are not present in current databases. Formally:

**Problem 1 [Shortcuts].** *Given a directed and weighted graph* 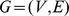 *and a set of sources* 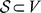 *and targets* 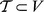 *, add* 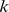 *edges to* 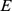 *to minimize* 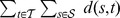 *, i.e. the total shortest-path distance between all source-target pairs.*

We use the shortest-path distance to measure the distance 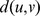 between proteins 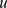 and 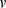 in the weighted network (as opposed to other distance measures, such as those based on random walks [44], [45]) because the shortest path represents a direct and specific series of high-likelihood signaling events.

The shortest path between two nodes in a weighted graph can be very long (either because the diameter is long or if the path uses many high confidence, and hence lowly weighted, edges). This may not be biologically reasonable since pathway targets are typically no more than 5 edges away from their closest sources [13]. Thus, we also propose a hop-restricted version of our problem. Let 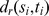 be the shortest-path distance between 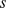 and 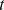 that uses at most 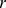 links (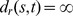 if no such satisfying path exists). Formally:

**Problem 2 [Shortcuts-X (restricted)].** *Given a directed and weighted graph* 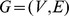 *, a set of sources* 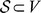 *and targets* 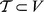 *, and a maximum allowable number of hops* 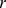 *, add* 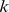 *edges to* 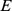 *to minimize* 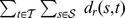 *, i.e. the total hop-restricted shortest-path distance between the pairs.*

Both of these problems (general and hop-restricted) assumes that each transcription factor receives signal from *each* source. Another variant of these problems asks to minimize the distance between each target and any single source (biologically, the same source does not need to regulate all targets, but every target is regulated by some source). Formally:

**Problem 3 [Shortcuts-SS (single source)].** *Given a directed and weighted graph* 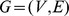 *and a set of sources* 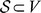 *and targets* 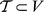 *, add* 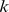 *edges to* 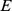 *to minimize* 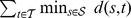 *, i.e. the total shortest-path distance between each target and its single closest source.*

We also consider the analogous problem in the hop-restricted setting:

**Problem 4 [Shortcuts-X-SS (restricted, single source)].** *Given a directed and weighted graph* 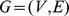 *, a set of sources* 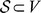 *and targets* 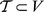 *, and a maximum allowable number of hops* 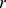 *, add* 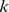 *edges to* 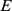 *to minimize* 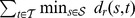 *, i.e. the total hop-restricted shortest-path distance between each target and its single closest source.*

In the Supporting Text (Text S1 and Figure S1) we prove that these four edge predictions problems are NP-hard.

### Greedy algorithm to predict pathway-consistent edges

Given these hardness results, we consider a heuristic greedy algorithm for our suite of edge prediction problems. The Greedy algorithm selects 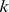 edges to add iteratively: in each step, it predicts a single edge that maximally reduces the objective function. In the case of the Shortcuts problem, this means the algorithm will pick, from amongst all possible non-existent edges, the edge that maximally reduces the global shortest-path distance between all sources and targets.

In a network with 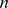 nodes and 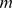 directed edges, there are 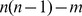 non-existent edges (excluding self-loops). In the yeast network we use, 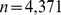 and 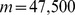, which means there are almost 20 million directed edges to test. Each edge can alter the shortest path from any source to any target hence, done navely, this would require recomputing the shortest-path lengths from each source to each target 20 million times just to add a single edge.

One trick to make the search more efficient is to notice that, if a candidate edge 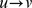 reduces the distance from source 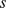 to target 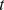 then the new shortest path from 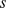 to 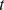 consists of three components: the shortest path from 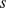 to 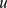, the candidate edge 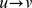, and the shortest path from 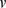 to 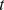. If it does not reduce the distance, then the distance from 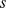 to 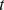 remains as it was without 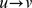. Thus, the procedure can be made more efficient by pre-computing the shortest-path distances from every source to every other node in the network, and separately from every node in the network to every target. (This latter step can be further optimized by computing the distance from every target to every other node in the reverse graph, where edge directions are reversed.) To compute the cost reduction of candidate edge 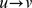 with weight 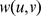 we check if:
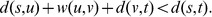
(1)


The left-hand side sums the (pre-computed) distance from 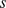 to 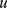, the weight of the new edge, and the distance from 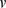 to 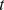; the right-hand side is the previous distance from 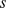 to 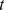 without the new edge. (If we do not know the weight of the non-existent edge we set 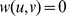 to encourage its usage; other values, e.g. based on the predicted likelihood of the 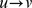 interaction that is derived from other data sources may also be reasonable). The minimum of these two values is stored and is summed over each source-target pair, yielding the new objective function cost assuming 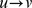 exists in the graph. The edge that maximally decreases the cost function over all possible edges is added to the graph. Box 1 shows the pseudocode for the Greedy algorithm for the Shortcuts problem.

**Box 1. Pseudocode of the Greedy Algorithm for the SHORTCUTS Objective**

**Table:** 

Algorithm 1. Greedy ( 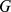 :directed graph, 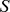 :sources, 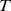 :targets, 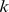 : number of edges to add)
1: 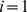
2: **while** 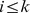 **do**
3: 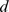 = source_target_shortest_paths_lengths( 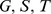 )
4: cost 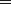 sum( 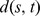 **for** 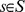 **for** 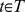 )
5: **for all** directed edges 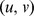 not in 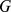 **do**
6: cost 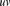 = sum(min( 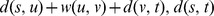 ) **for** 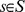 **for** 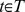 )
7: **if** cost 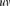 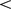 cost **then**
8: cost = cost 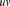
9: 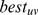 = 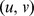
10: **end if**
11: **end for**
12: add edge 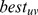 to 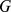
13: **end while**

For the Shortcuts-SS problem, line 6 of the algorithm is modified to compute the sum of distances from each target to its single closest source. This way, each target is modeled to be regulated by one source as opposed to every source.

This trick reduces the algorithm's complexity in each step from 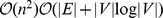 in the nave case to 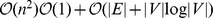. The first term considers all possible non-existing edges, each of which requires a constant lookup (Equation 1); the second term is the pre-computation of single-source shortest-path distances using Dijkstra's algorithm. Thus, we get a runtime reduction of a factor of 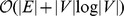, which in our case is roughly 60,000 for each iteration.

### The hop-restricted greedy algorithm

For the hop-restricted problems (Shortcuts-X and Shortcuts-X-SS), we seek short paths between sources and targets with the restriction that each path uses a maximum of 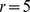 hops. This bound stems from the fact that many pathways in signaling databases such as KEGG [46] depict on average 5 edges between a target and its closest source [13]. Other approaches have used similar bounds (3–4 [47]).

To constrain the shortest paths to use at most 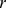 edges, we use a modified version of the Bellman-Ford algorithm [48], [49]. This algorithm computes single-source shortest paths starting from a node 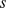 by relaxing every edge in each step (i.e. checking if traveling along the edge yields a shorter path to the destination node). Shortest-path distances are propagated through the graph and, as a result, after 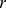 iterations, the algorithm computes the shortest-path distance from 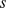 to every other node in the graph using at most 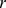 hops.

Computing the updated cost for a candidate edge, however, requires a slightly different strategy than the one used before. The main challenge is that the new edge 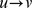 induces one hop, and hence, the two sub-cases (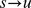 and 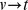) must be constrained to use 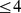 hops in total. This leads to 6 possible cases to consider for the each candidate edge 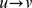 when computing the new distance from source 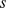 to target 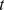, and each can be computed in constant time:
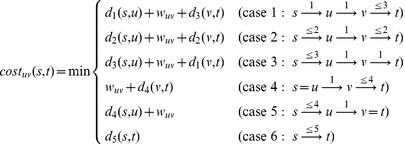
(2)In the first case, the new path from 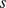 to 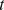 uses 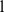 hop to reach 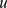, 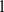 hop to reach 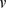 (via the new edge), and 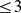 hops to reach 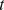. The cost of this path consists of the Bellman-Ford distances shown (where e.g. 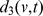 is the distance from 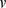 to 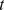 that uses at most 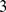 hops) plus the weight of the new edge (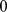). Cases 2 and 3 follow similarly. If either endpoint of the candidate edge involves 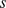 or 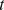, then a similar rule is checked (cases 4 and 5). Each case is considered and the one that yields the minimum distance is compared with the previous distance from 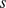 to 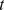 (without the new edge; case 6). For the Shortcuts-X problem, this is repeated for each source-target pair; for Shortcuts-X-SS this is done for each target to find the hop-restricted distance to its closest source.

After an edge is added, the Bellman-Ford distances are re-computed (from sources to all nodes in the graph and from targets to all nodes in the reversed graph) and the process is repeated greedily. This algorithm takes time 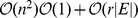 per step. The first term evaluates the benefit of each possible edge (Equation 2); the second term is the pre-computation of single-source hop-restricted shortest-path distances using the Bellman-Ford algorithm.

### Computational experimental setup

#### Network

We used a protein-protein interaction (PPI) network for *S. cerevisiae* compiled from the STRING database of known and predicted protein interactions (v9.0) [50]. We only consider known physical binding interactions (excluding protein-DNA interactions), each of which is further weighted based on evidence from high-throughput experiments, genomic context, co-expression, and text mining. These weights allow us to implicitly incorporate a wide variety of biological features into our framework. All weights 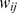 are transformed to 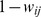 so that higher confidence edges imply shorter paths. The original network contained 5,874 proteins and 55,623 interactions (Table 1) though some of these nodes and interactions were not used in the final oriented network (see below).

**Table 1:** Data and statistics.

STRING PPI network	Oriented network	Sources	Targets		
5,874 proteins	4,371 proteins	Cdc42	Cin5	Hot1	Mcm1
55,623 physical interactions	47,500 interactions	Hkr1	Msn1	Msn2	Msn4
659,717 potential interactions		Msb2	Skn7	Sko1	Smp1
		Opy2	Sok2	Yap6	
		Sln1			

The undirected protein interaction network from STRING contained 55,623 interactions amongst 5,874 proteins. Starting from this network, the orientation algorithm purged 1,503 proteins and 8,123 edges that were not on any 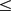
5-hop path between a source and target pair. Of the almost 20 million non-existing edges, STRING provided evidence for 659,717 potential edges that were each weighted by a confidence value in [0,1]. We included every potential edge that had weight 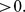 We used 5 sources and 11 targets.

#### Pathway sources and targets

We focused on the HOG MAPK signaling pathway, known for its role in osmotic stress response in budding yeast [51], [52]. Sources were chosen as upstream proteins that had no incoming edges in the pathway according to KEGG [46], the Science Signaling *Database of Cell Signaling* [53], and de Nadal and Posas [54]. Targets included the core HOG pathway transcription factors (TFs) as well as secondary TFs implicated in osmotic stress response [46], [52], [53], [55]. The 5 sources and 11 targets we use are shown in Table 1.

#### Orienting the network

Although protein interactions deposited in databases (such as STRING) are usually undirected, pathways interactions often have a strict directionality. Recently, Gitter et al. [13] proposed an algorithm to discover putative pathways embedded within undirected interaction networks. Their method orients edges in the network to maximize the number of weighted, hop-restricted paths between a given set of sources and targets, and it was shown to successfully extract pathways in yeast. We used this algorithm to orient the STRING PPI network using the sources and targets mentioned above and with a hop-bound of 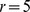. The corresponding oriented network contained 4,371 proteins and 47,500 directed interactions (Table 1). Note that our framework does not necessarily require directed edges, but we use them to more realistically model signaling pathways in the cell.

To quantify the correctness of the predicted edge directions, we computed the percentage of KEGG and Science Signaling HOG pathway edges that were oriented correctly. Of the 16 KEGG edges, 9 existed in the STRING PPI network and 7 of these (77.8%) were oriented correctly. Similarly, of the 42 Science Signaling edges, 29 existed in the STRING PPI network and 18 of these (62.1%) were oriented correctly. Thus, while some errors were likely made by the orientation step, a substantial portion of the edges were directed appropriately.

### Other algorithms to predict missing interactions

We compare our Greedy algorithm to several other popular algorithms for predicting missing interactions.

#### Direct-ST

This method only predicts direct edges from sources to targets. For each of the four problems, this algorithm will predict the source-target edge that maximally reduces the respective cost function.

#### Betweenness

A natural and intuitive algorithm is to predict edges that lie highly “central” to the sources and targets. The *betweenness centrality* of an edge is defined to be the number of all-pair shortest paths that use the edge. Edges that have high betweenness centrality can be thought of as bottleneck or bridge edges that efficiently connect two parts of the graph. Tastan et al. [56] trained a classifier to predict host-pathogen interactions and (node) betweenness emerged as a high-weight feature. In our case, we compute the betweenness centrality of each non-existent edge (assuming it were added to the graph), and instead of summing over all pairs of nodes in the graph, we only consider source-target pairs. Thus, in each step we add the non-existent edge that has the highest centrality between the sources and targets. For Shortcuts-SS, an edge is considered used if it helps reduce the distance between a target and its single closest source. Note that the usage of an edge when computing the betweenness centrality is a binary value 0 or 1, and this algorithm does not explicitly take the magnitude of cost reduction into account. The Betweenness algorithm is similarly adapted in the hop-restricted case to use the Bellman-Ford distances. For example, for Shortcuts-X, we add the edge that is used by the most hop-restricted shortest paths between sources and targets.

We also compare to two global methods that do not leverage the sources and targets directly:

#### Jaccard

One popular approach to predict new edges is based on shared interaction neighborhoods. If non-interacting nodes 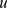 and 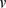 share many common neighbors, this implies a similar functional role and therefore a likely interaction. This general principle has been used by many function- and edge-prediction pipelines in the literature [33], [35], [37].

To adapt this measure for weighted graphs, we compute the weighted Jaccard coefficient between (non-interacting) proteins 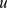 and 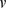 as the sum of the weights to shared neighbors of 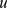 and 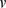 divided by the total sum of neighbor weights for each protein. We also multiply this ratio by the number of common neighbors so that proteins with more common neighbors are biased towards. For all four problems, in each iteration, we add an edge between the two proteins with the highest weighted Jaccard coefficient.

#### Short-Path

The shortest-path distance between two proteins has also been used in various contexts to predict putative interactions and functional relations of the two proteins [37], [57]–[60]. For our problems, in each iteration, we add the edge connecting the two closest (but non-interacting) proteins in the network.

In all algorithms (including Greedy), ties are stored and picked from randomly.

### Computational validation of predicted interactions

Several strategies have previously been used to validate network-based edge predictions [34], [61]. First, we describe the notion of *potential edges*, and then we describe four validation techniques using these edges.

The STRING database aggregates protein-protein associations from over a dozen other pathway and protein interaction databases and combines these with computational predictions based on sequence, co-expression, literature mining, interactions between orthologous proteins, and other biological features to provide a comprehensive protein relationship resource [50]. Only a small subset of these relationships, however, represent physical binding interactions. The remainder, which we term *potential edges*, are composed of other types of experimentally- or computationally-derived non-physical associations. STRING assigns edge weights for both types of edges (physical and potential) based on biological and computational evidence supporting the link. One benefit of the STRING weighting scheme is that weights for both the physical and potential edges are computed in the same manner and thus are directly comparable. Edges supported by multiple types of evidence have higher weights [62]. Our predictions are based solely on the network topology and source-target connectivity — they do not rely on sequence, gene expression, or any of the other data types — and are therefore completely independent of the STRING predictions.

Starting from only the STRING physical interactions, one way to test our predicted edges is to count how many of them exist within the set of STRING potential edges. The STRING potential network contains 659,719 of the approximately 20 million possible interactions (3.5%), hence identifying the correct interactions is still very challenging.

Although identifying STRING potential edges is useful, these predictions may not bear any relevance to the HOG pathway from which the sources and targets are derived. Our second validation approach considers a prediction as correct if it exists within the STRING potential edges *and* it connects two proteins from the set of sources, targets, and other known HOG pathway members [46], [53]; otherwise it is incorrect. KEGG and the Science Signaling *Database of Cell Signaling* provide an unbiased set of pathway members that are not dependent on our own subjective curation efforts. Although these pathway databases omit some HOG members reported in recent literature (e.g. the upstream proteins in de Nadal and Posas [54]) and other uncharacterized proteins that partake in the osmotic stress response, the proteins and interactions they do contain are provided by pathway experts and are thus trustworthy. Therefore this test serves as a strong proxy for each method's ability to make high quality and pathway-relevant predictions.

Our third test measures the quality of an edge prediction based on how much its addition reduces the objective function cost. This approach directly quantifies the method's ability to reduce the distance between sources and targets.

Finally, as a fourth test, we conducted the following cross-validation experiment: We started with the unoriented STRING PPI network and identified all the edges connected to at least one HOG-relevant node (there were 1079 such edges). Because our algorithm specifically predicts edges that lie between sources and targets, these HOG-related edges were used as the cross-validation set. We performed 5-fold cross-validation for the Greedy algorithm using the Shortcuts and Shortcuts-X objective functions and counted how many of the top 10 predictions exactly recovered a left-out edge. The probability that a random prediction would recover a left-out edge from amongst all the potential edges is extremely small (0.033%), and thus this test is also very challenging. It is also challenging because it is difficult to decouple training and test sets of edges. Leaving out even a very small number of edges may result in an entirely different pathway structure in which alternative paths may emerge as more likely. This is especially prevalent on small scales: for example, if edges 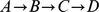 exist and the edge 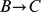 is left-out, then it is entirely reasonable to predict edge 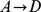 as a shortcut of the path chain. More generally, any chain can be shortcutted by directly connecting the ends (which may often be hubs through which the paths diverge), and single-use edges that play a peripheral role in the pathway may be bypassed altogether.

To summarize, we consider four approaches to validate edge predictions. The first test compares the prediction accuracy of each method in identifying STRING potential edges. The second test compares the prediction accuracy of each method when predicting STRING potential edges that are also relevant to the HOG pathway. The third compares each method's ability to reduce the objective function cost. And the fourth measures the cross-validation accuracy of the Greedy algorithm.

## Results

We started with sets of HOG pathway sources and targets and an undirected, weighted PPI network for *S. cerevisiae* from STRING composed of only physical binding edges (Table 1). We oriented the network [13] and used the three source-target-based algorithms (Greedy, Betweenness, Direct-ST) and two global algorithms (Jaccard, Short-Path) to predict directed edges in this network using the relevant objective functions (Shortcuts, Shortcuts-X, Shortcuts-SS, Shortcuts-X-SS). We evaluated each method with respect to its ability to: 1) reduce the objective function cost; 2) predict edges that lie within the STRING potential edges; and 3) predict edges that lie within the STRING potential edges that also connect known HOG-related nodes. For the Greedy method, we also performed cross-validation experiments.

### The Greedy algorithm drastically reduces source-target distances

**Figure 2:**
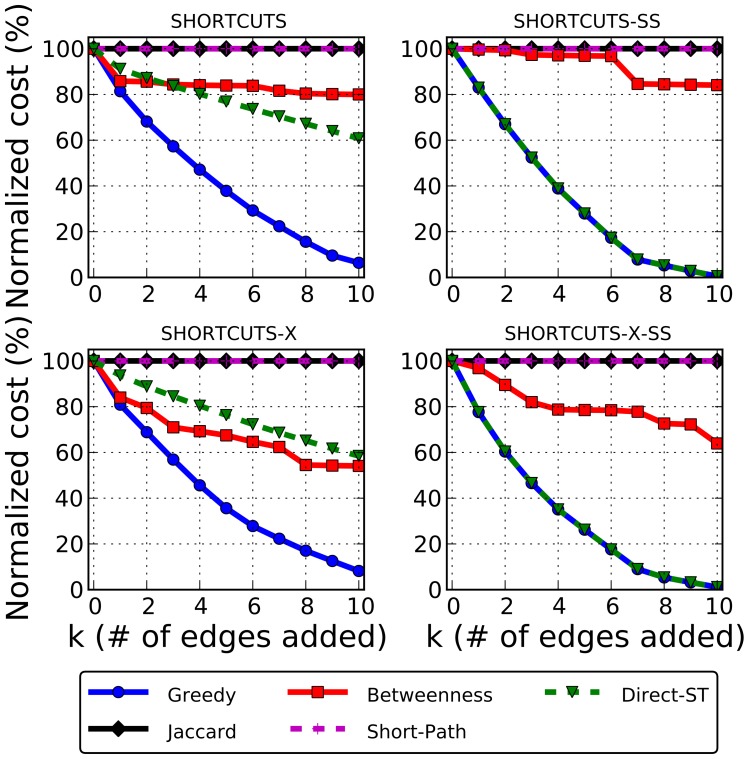
The cost reduction achieved by the five methods for each objective function. The 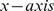 shows the number of edges added, and the 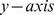 shows the new objective function cost as a percent of the original cost. Each new edge was added with weight 0.0. For Shortcuts and Shortcuts-X, Greedy significantly outperforms all other methods. For Shortcuts-SS and Shortcuts-X-SS, both Greedy and Direct-ST perform equally. As expected, the global methods (Jaccard and Short-Path) select HOG-independent edges that do not reduce any source-target distances.

Our Greedy algorithm achieves the greatest cost reduction compared to the other four methods over all variants of the pathway-aware edge prediction problems (Figure 2). Moreover, Greedy substantially decreased source-target distances after adding only a few edges. For example, after adding 3 edges, the Shortcuts cost (measured as the total shortest-path distance amongst 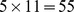 source-target paths) can be reduced to approximately 60% of the original cost. In contrast, it takes 10 edges for Direct-ST to achieve the same ratio. The Betweenness algorithm does monotonically decrease the cost, however, because edges are added based on greater usage (as opposed to greater explicit cost reduction), its reduction is much slower than Greedy overall. The global methods (Jaccard and Short-Path) do not leverage the sources and targets and therefore are unable to reduce source-target distances at all; in general, there are an enormous number of possible edges that play no putative role in the pathway and it is difficult for these methods to disambiguate these edges from HOG-relevant edges. The tremendous cost reduction seen with the Greedy predictions implies that there are a few missing edges in the network whose addition may cover a large bulk of the information flow in the network.

For Shortcuts-SS and Shortcuts-X-SS, both Greedy and Direct-ST perform equally well. This is because there are only 11 paths to optimize over instead of 55 (each target to a single source). Thus, a viable strategy is to find the target 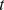 that is furthest away from any source and connect a source directly to it. This can greatly reduce the cost function, even if no other path uses this edge, though this need not be the case in general.

### Comparing the prediction accuracy of each method

**Figure 3:**
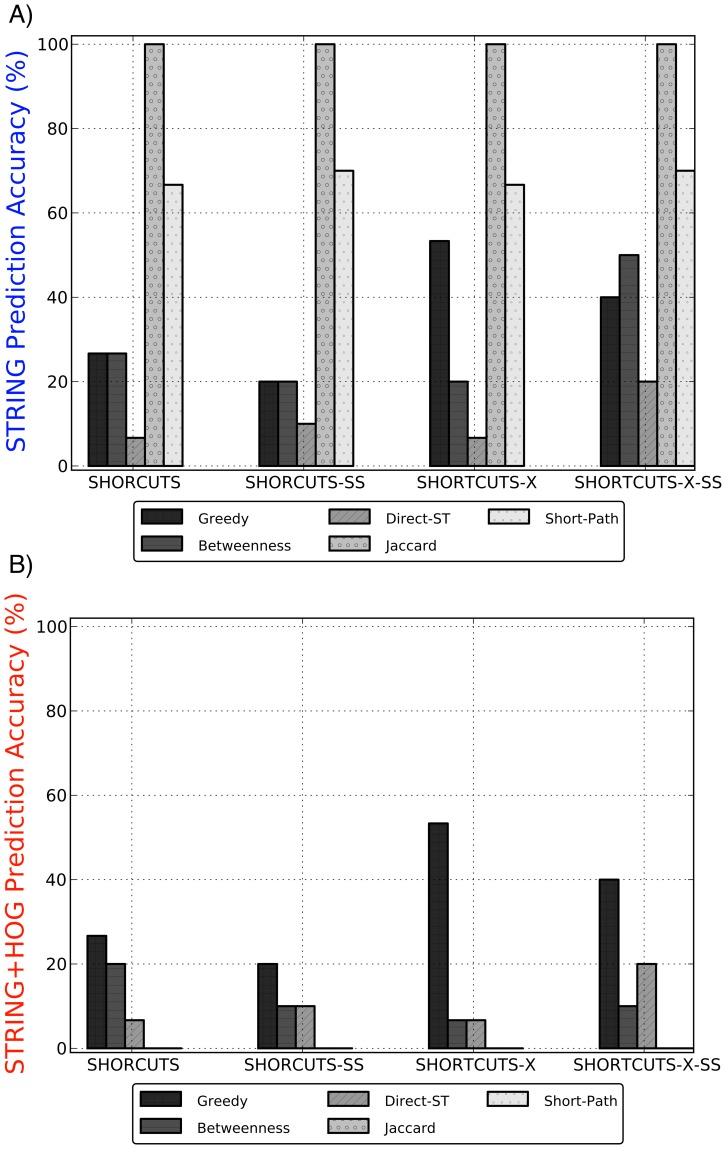
The prediction accuracy of the five methods for each objective function. We evaluated the top 15 (Shortcuts and Shortcuts-X) or 10 (Shortcuts-SS and Shortcuts-X-SS) predictions for each algorithm, after which the Greedy algorithm had reduced the objective function to nearly zero. The 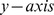 shows the prediction accuracy, defined as the percentage of predictions (from amongst all 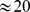 million possible missing edges) that lied within the set of A) STRING potential edges, and B) STRING potential edges that also connected known HOG-related proteins. The global methods (Jaccard and Short-Path) make accurate predictions when not constrained to be HOG-relevant. The Greedy algorithm outperforms all methods in making high quality predictions that connect HOG proteins.

Next, we judged the quality of the predictions based on how well they overlapped with the STRING potential edges and with HOG-relevant proteins (Figure 3). In these tests, the accuracy of the method is the percentage of predicted edges, made from amongst all possible non-existent edges, that lied in the relevant set.

When only considering support in STRING (Figure 3A), we find that the global methods (Jaccard and Short-Path) significantly outperform the source-target-based methods. In particular, every prediction made by the Jaccard algorithm is correct according to STRING as are over 60% of the Short-Path predictions. This result agrees with previous studies that showed that network distance and shared topology are strong indicators for functional or physical relatedness [33], [35], [37], [57]–[59]. The probability of predicting a STRING potential edge from amongst all possible edges is only 3.5%, and thus most approaches perform significantly better than baseline.

This test, however, does not tell us whether the predictions bear any relevance to the HOG pathway, which is the primary focus of this study. To better home-in on HOG-relevant predictions, we filtered the STRING potential edges to only include those edges that connected two known HOG-related proteins. Figure 3B shows that the global methods do not make any predictions that relate to the HOG pathway. On the other hand, the Greedy predictions remain at the same level in both tests, which implies that its predictions tend to be highly accurate *and* lie amongst HOG-related nodes. The difference is especially pronounced in the hop-restricted cases, where Greedy is more accurate than any other method by roughly 40% (Shortcuts-X). Two of these edges connect Hog1 to known HOG transcription factors, Msn4 and Cin5 — both previously established interactions in KEGG [46] or the literature [63] (which are missing from the STRING database and thus do not appear in the original network we used). The probability of predicting a HOG-relevant STRING potential edge from amongst all possible edges is only 0.076%, which is much lower than the accuracy of all three source-target-based algorithms.

Of the top 15 predictions made by Greedy and Betweenness for the Shortcuts-X problem, only one prediction overlaps, and a similar trend holds for the other objectives. This likely stems from the fact that Greedy takes the magnitude of the cost reduction into account, whereas Betweenness only computes the number of shortest paths that use the candidate edge. Because both algorithms perform significantly better than baseline, this implies that they may provide complementary predictions and both may be reasonable depending on the use case.

Interestingly, despite their similar performance in cost reduction for Shortcuts-SS and Shortcuts-X-SS (Figure 2), Greedy makes more accurate predictions than Direct-ST (Figure 3). This is because there are many cases where a direct source-to-target prediction can be equivalently replaced by a target-target interaction. For example, if 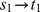 was added in the first step, the predictions 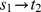 and 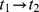 (regulated via 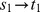) both equally reduce the cost from a single source (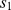) to the target 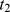. However, target-target interactions are more likely to exist within the STRING potential edges than direct source-target edges, and indeed Greedy makes several TF-TF predictions (e.g. 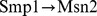), thereby giving it an advantage.

To show that the orientation step is indeed useful in extracting HOG paths given sources and targets, we ran each algorithm on the *unoriented* STRING PPI network (Figure S2). We found that for both hop-restricted objective functions, the Greedy algorithm makes more HOG-relevant predictions when using the oriented network (53% vs. 46% for Shortcuts-X and 40% vs. 20% for Shortcuts-X-SS, compared to using the unoriented network). Moreover, the global methods (Short-Path and Jaccard) also benefited significantly from the orientation, which implies that defining network neighbors more precisely can help in identifying putative interactions.

Overall, these results show that the global methods perform well in identifying putative interactions, but that the Greedy algorithm can home-in on more pathway-consistent interactions while drastically reducing source-target distances.

### Integrating additional biological features into the framework

While predicting plausible edges from amongst all possible edges serves as a strong validation technique, in practice, we would also like to leverage other data sources (such as expression, sequence, and literature evidence) when making predictions. To naturally integrate these sources into our framework, instead of predicting from amongst all possible edges, we only predict from amongst the set of STRING potential edges (Methods). Each potential edge is weighted by STRING with a confidence value in 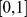, which we explicitly set to 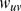 (Equations 1 and 2; in the previous sections, 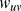 was given a default weight of 0). By using these data types and weights together, we can pinpoint putative interactions that have evidence from a wide variety of biological sources as well as evidence from the network.

Table 2 presents the top 10 predictions made by the Greedy algorithm for the Shortcuts objective function, many of which are known physical interactions missing from STRING. The 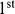 and 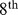 predictions have direct evidence of physical interaction according to BiOGRID [64], but were not present in the STRING network. The 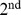 and 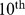 predictions lied within the STRING binding edges (and thus represent physical interactions), but were either oriented in the opposite direction or were left out of the oriented network. 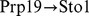 was originally oriented 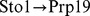, but the Greedy algorithm suggests that that this edge was either oriented incorrectly or is bidirectional. 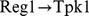 was left out of the network because the orientation algorithm did not find any length-bounded paths that included this edge. Although in general biological pathways are short, this prediction exemplifies an exception where considering longer pathways through the edge 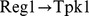 improves the source-target connectivity. These correct predictions demonstrate that our approach can correct for limitations of the edge orientation.

**Table 2:** Top 10 predictions for Shortcuts using the Greedy algorithm.

#	Src	Tgt	Score	Weight	Comments
0	—	—	12.91	—	Original objective function cost
1	Hkr1(s)	Syf1	11.63	0.998	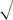 Physical interaction in BioGRID [PCA high-throughput]
2	Prp19	Sto1	10.13	0.999	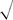 Oriented in opposite direction; BioGRID [Affinity Capture-MS]
3	Ssk1	Sho1	9.12	0.999	Only indirect interaction reported; two different HOG input paths
4	Tpk2	Sok2(t)	8.19	0.996	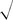 We studied experimentally [see Results and Discussion]
5	Reg1	Msn4(t)	7.35	0.999	Indirect partners; both physically interact with Bmh1/2 [66]
6	Msn4(t)	Msn2(t)	6.63	0.999	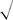 Msn4 binds Msn2 in succinic acid [65]
7	Hog1	Cin5(t)	6.06	0.872	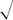 Hog1 binds Cin5 in osmotic stress [63]
8	Bem2	Cdc42(s)	5.72	0.998	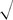 Physical interaction reported in BioGRID [Biochemical activity]
9	Msb3	Yap6(t)	4.93	0.915	Only indirect interaction reported
10	Reg1	Tpk1	4.77	0.999	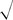 STRING binding edge, but left out of orientation

The original value of the objective function (score) was 12.91. The *Src* and *Tgt* columns indicate the direction of the predicted edge. The markers (s) and (t) imply that the protein was an original HOG source or target, respectively. The weight of the edge comes from STRING. Predictions for which there is evidence of direct, physical interaction are shown with a checkmark.

For the following three predictions, we verified both the physical interaction between the two nodes and the directionality (which is not possible for edges validated with the undirected STRING or BioGRID databases). The 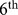 prediction (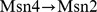) involves two general stress TFs that play a substantial role in the HOG pathway [51]. Harbison et al. [65] showed that indeed Msn4 binds the *MSN2* gene in the succinic acid stress condition. This study did not profile Msn4 DNA binding in osmotic stress, but it is plausible that this stress-activated TF could bind *MSN2* in other conditions as well. The 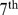 prediction (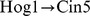) was recently shown by Pokholok et al. [63] to occur in osmotic stress. We discuss the 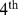 prediction (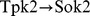) at length in the next section.

Overall, 7 of the top 10 predictions have support for direct physical binding in the cell. In addition, the 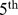 prediction was not directly supported in the literature but warrants further study. Both Reg1 and Msn4 have been shown to physically associate with the 14-3-3 proteins Bmh1 and Bmh2 [66] but have not yet been shown to directly interact with one another. Proteins with a common physical interaction partner may be more likely to directly interact themselves than proteins with other types of functional connections (e.g. genetic interactions) [33], [35], [57].

Table 3 presents the top 10 predictions made by the Greedy algorithm for the Shortcuts-X objective function, which attempts to model more biological constraints by imposing a hop-restriction on the source-target paths. Remarkably, the top three predictions (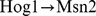, 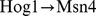, and 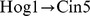) represent best-case predictions: The two genes/proteins involved are known to physically interact, the directionality is correct, and the interaction is highly relevant to osmotic stress response. In particular, 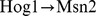 and 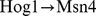 are core HOG pathway interactions that are well-characterized [51] and appear in KEGG [46], but lack evidence for physical binding in STRING. The MAPK Hog1 is central to the HOG response program, and its activation of downstream TFs is a critical component of the response. The other two validated predictions involve HOG pathway members as well. Sho1 is a transmembrane osmosensor, and its branch of activation of Hog1 is known to be mediated by interaction with Cdc42 [67]. The 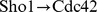 interaction is also present as part of the related starvation subpathway of MAPK in KEGG [46]. Finally, the 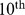 prediction (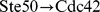) is between two members of the Sho1 HOG pathway input branch [53]. Overall, of the 659,719 STRING potential edges considered, only 0.0011% are in KEGG, and thus the fact that 3 of the top 10 predicted edges lie in KEGG is highly significant (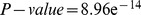, Fisher's exact test).

**Table 3:** Top 10 predictions for Shortcuts-X using the Greedy algorithm.

#	Src	Tgt	Score	Weight	Comments
0	—	—	18.24	—	Original objective function cost
1	Hog1	Msn2(t)	15.93	0.968	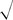 Hog1 activates Msn2 in osmotic stress [51]; KEGG
2	Hog1	Msn4(t)	14.34	0.962	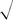 Hog1 activates Msn4 in osmotic stress [51]; KEGG
3	Hog1	Cin5(t)	12.76	0.872	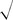 Hog1 binds Cin5 in osmotic stress [63]
4	Hkr1(s)	Ste20	11.96	0.802	Only indirect interaction reported
5	Sln1(s)	Ptc1	11.31	0.968	Only indirect interaction reported
6	Msb3	Yap6(t)	10.82	0.925	Only indirect interaction reported
7	Sho1	Cdc42(s)	10.08	0.965	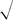 Cdc42 required for Sho1-activation of Hog1 [67]; KEGG
8	Sln1(s)	Sho1	9.72	0.959	Only indirect interaction reported; two different HOG input paths
9	Cla4	Swi4	9.32	0.983	Only indirect interaction reported
10	Ste50	Cdc42(s)	8.64	0.989	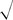 Oriented in opposite direction; BioGRID[Complex, Y2H]

The original value of the objective function (score) was 18.24. The *Src* and *Tgt* columns indicate the direction of the predicted edge. The markers (s) and (t) imply that the protein was an original HOG source or target, respectively. The weight of the edge comes from STRING. Predictions for which there is evidence of direct, physical interaction are shown with a checkmark.

Other predictions whose physical interaction could not be validated also involve pairs of HOG pathway members. Some predictions occur between the two independent upstream input branches in the pathway (e.g. 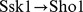 and 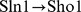) or between upstream proteins and proteins that are very far downstream (e.g. 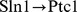). From an algorithmic standpoint, these edges do indeed provide faster diffusion of signal from sources to targets; however, they may not represent direct interactions that occur in the cell. In contrast, the 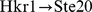 prediction is a shortcut within the Sho1 input branch, which contains the cascade 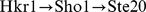 [54]. Note that several of these predicted edges have very high weights (e.g. 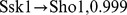) from STRING reflecting their strong functional dependencies, which makes them more likely to be selected by our algorithm. However, several predictions were made despite lower evidence (e.g. 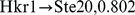), which suggests that their addition strongly aided source-target connectivity. Interestingly, none of the top 10 predictions directly connects a source to a target. This further necessitates an approach like ours versus Direct-ST.

To further validate our ability to extract accurate pathway-relevant predictions from within the potential set, we conducted 5-fold cross-validation experiments by leaving out HOG-relevant edges (see Methods). The probability that a random prediction would recover a left-out edge from amongst all the potential edges is extremely small (0.033%). Using the Greedy algorithm, we found that 12% (16%) of the top 10 predictions for Shortcuts (Shortcuts-X) recovered a left-out edge. Recovering one correct edge (10%) yields a P-value of 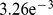 and recovering two correct edges (20%) yields a P-value of 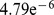 (Fisher's exact test). Both values are significant (our results lie between them) further supporting the ability of our method to make accurate edge predictions.

To explore the sensitivity of our results to the hop-restriction length, we repeated our computational experiments using a hop-restriction length of 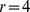. Overall, we found similar qualitative performance for the algorithms when predicting from amongst all possible edges (Figure S3). However, when predicting from amongst the potential set, we found only a few overlapping predictions with those made when the hop length was 5. Interestingly, these included the well-known HOG interactions 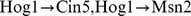, and 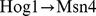, suggesting that the most confident and likely predictions are not wholly affected by the decreased hop restriction. Of course, some different predictions are also to be expected; for example, using a hop length of 4, the algorithm makes predictions for 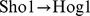 and 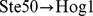. While these predictions make sense algorithmically, they do not make sense biologically because they attempt to shortcut the sources of the pathway directly to a core node (Hog1). This suggests that 4 hops may be too restrictive and may motivate using a hop restriction of 5 in future efforts.

We also found that our approach was able to recover missing interactions when not leveraging the STRING-derived weights (see Text S1). This implies that our approach is not entirely dependent on the potential edge weights and that our objectives are well-defined.

### 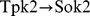: A novel prediction

To demonstrate our approach's ability to make novel, biologically meaningful predictions we selected 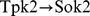 for experimental validation. This was a top prediction for two objective functions (for Shortcuts-SS it was the 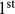 prediction and for Shortcuts it was the 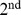 uncharacterized prediction; Table 2). As we showed, the addition of a few edges can greatly reduce the objective function cost, and therefore we place more confidence in these top edges.

Verifying a directed protein-protein interaction at the mechanistic level requires extensive experimentation and is beyond the scope of this work. However, genetic experiments such as gene deletions can establish condition-specific causal relationships between proteins in signaling pathways. For instance, loss-of-function mutations and gene over-expression were used to identify and order the genes along the apoptosis pathway in *C. elegans* [68]. In our case, if Tpk2 controls the TF Sok2 in osmotic stress, *TPK2* deletion should affect Sok2's regulatory activity in this condition. Because many interactions along signaling pathways occur post-translationally, we would not expect the *SOK2* gene to be differentially expressed in the 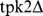 mutant even if Tpk2 does activate or inhibit Sok2 at the protein level. Instead we determine the degree to which the deletion alters Sok2's function as a transcriptional regulator. As predicted, the knockout significantly affected genes bound by Sok2 (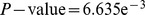, Fisher's exact test; see Supporting Text S1 for microarray details and Table S1 for lists of affected genes). The knockout alone cannot confirm whether the 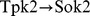 interaction is direct or indirect, but clearly establishes that there is a functional connection between these proteins that is active in osmotic stress. Moreover, the orientation of the predicted 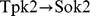 edge is correct because if Sok2 were upstream of Tpk2 in the pathway, its bound genes would be unaffected by *TPK2* deletion.

To test the significance of our knockout (KO) with other perturbation experiments, we used the Rosetta compendium [69] of 300 KO expression experiments and compared the overlap between differentially expressed (DE) genes in each experiment with the list of Sok2 targets (see Supporting Text S1). Of 301 experiments, only 31 (10.3%) had a lower P-value than the one obtained from our *TPK2* KO. In the other direction, we considered 117 additional TFs for which a high confidence set of targets exists [70]. For each, we computed the significance of the intersection between their targets and genes affected by the *TPK2* deletion using Fisher's exact test. Similar as the test above, of the 118 tests only 14 (11.9%) had a lower P-value than our predicted Tpk2-Sok2 pair. Combined, our predicted interaction ranked close to the top 10% in these two independent analyses further supporting our prediction.

## Discussion

Protein interaction networks encode a variety of signaling processes that occur in the cell, however, many interactions are still missing and experimental validation of all putative interactions is unlikely in the near future. This has led to a proliferation of computational methods to aid in identifying putative interactions. One particularly important task when mining these networks is to identify pathways. Experimental protocols have made it possible to identify upstream proteins that trigger information cascades to downstream transcription factors. Many techniques have been proposed to extract likely subnetworks from within global interaction networks, however, these approaches do not formally model interactions that are missing from the network.

We presented a new framework for predicting missing edges that lie “in-between” given sets of sources and targets within the network. Compared to four other edge prediction algorithms, our Greedy algorithm was able to home-in on more pathway-consistent interactions while substantially reducing source-target distances by only adding a few edges. We also showed how to naturally integrate other biological features into the pipeline and used this evidence to recapitulate many known but missing physical interactions, including several interactions reported in KEGG and other databases and reports.

Our ability to correctly predict context-specific directed PPIs by reducing source-target distances with the Greedy algorithm yields high-level biological insights into signaling network topology. In many cases the endpoints of a predicted edge are already connected via a longer alternate pathway. Shortcut edges between connected proteins form alternate paths for signal flow, which may lead to a greater degree of robustness in the pathway. In addition, such edges may indicate that the two proteins are participating in a feed-forward loop. The feed-forward loop motif can provide precise control of activity timing and noise filtering [71] so recognizing that a pair of proteins belong to a feed-forward loop instead of a linear chain improves our understanding of their role in the signaling pathway. Our objective functions encourage adding edges that reduce the distance between multiple source-target pairs, and indeed, we find that the first few predictions (those that improve the objective function the most) when using the Shortcuts or Shortcuts-X objective benefit many such pairs. For Shortcuts, the first 3 added edges decrease the distance of 27 of the 55 source-target pairs (49.1%). Likewise, the first 3 Shortcuts-X predictions reduce the distance for 18 pairs (32.7%). These first few predictions are also highly accurate (Tables 2 and 3), indicating that edge-reuse is an important principle in signaling networks.

In general, the predictions varied as more constraints were added to the objective function: with respect to Shortcuts, 50% of the top 10 predictions overlapped with Shortcuts-SS and only 20% with Shortcuts-X and Shortcuts-X-SS. Initially, without any hop-restriction, the average number of hops to connect a source and target is 7.8 (with total distance 12.91). When applying the 5-hop-restriction (as in Shortcuts-X), alternative edges are forcibly used that have lower confidence, and thus the total distance increases to 18.24. The hop-restricted objectives thus lead to a restructuring of the source-target paths and tend to select central nodes through which much signal flows (e.g. Hog1). The non-hop-restricted algorithms may induce alternative longer paths that circumvent these hubs. This implies that there is a trade-off between the likelihood of a series of interactions (the weights along the path) and the efficiency of the source-target cascade (the number of hops along the path). The former is characterized by the Shortcuts objective, while the latter is captured by Shortcuts-X. While evidence exists supporting predictions from both objectives, the hop-restricted versions found more predictions that were actually in the KEGG HOG pathway (3 versus 0) and that connected two known HOG pathway members (8 versus 3; compare Tables 2 and 3). This suggests that Shortcuts-X predictions may have greater fidelity with the condition-specific pathway (which is our focus here). On the other hand, Shortcuts made more predictions whose physical binding could be verified than Shortcuts-X (7 versus 5), which suggests that this objective may be capturing more general interactions that aid overall network connectivity.

### The role of Tpk2 and Sok2 in the osmotic stress response

Our knockout experiment examines the predicted relationships between Tpk2 and the target TF Sok2 in hyperosmotic stress conditions. Tpk1, Tpk2, and Tpk3 form the catalytic subunit of protein kinase A (PKA), the complex at the heart of the Ras/cAMP/PKA signaling pathway [72]. Through interactions with its many substrates, PKA is involved in general stress response, metabolism, growth, ribosome biogenesis, and various other biological processes [72], including osmotic stress response. PKA's involvement in the osmotic stress response is parallel to the HOG pathway [73]. Msn2, Msn4, and Sko1, which along with Hot1 are considered to be the primary HOG pathway TFs [51], are each affected by PKA in osmotic stress [73], [74]. Decreased PKA activity modulates the repressive effects of Sko1 in this condition. This behavior is complementary to Hog1's phosphorylation of Sko1, which also alleviates Sko1 repression of its target genes [73]. While Tpk2's role in osmotic stress is well-established, Sok2 is not considered to be a core HOG pathway TF, but was rather assumed to be controlled by the primary TFs [52]. However, genetic screens illustrate that its role in the osmotic stress response may be larger [75], [76].

Our *TPK2* knockout establishes a functional link between Tpk2 and Sok2 in which Sok2 is downstream of Tpk2. A previous genetic interaction reported by Ward et al., who suggested that PKA may directly phophorylate Sok2, supports this directionality and relationship [77]. Subsequent experiments confirmed that active PKA phosphorylates Sok2 when glucose is the carbon source [78]. However, this link does not appear in other conditions. For example, Sok2 was found to function in a pathway parallel to PKA [79] and Tpk2 [80] in pseudohyphal growth and adhesive growth, respectively. In addition, Tpk2 does not interact with Sok2 in a mutant yeast strain that is sensitive to exogenous cAMP [81]. These findings highlight the importance of pathway-specific predictions of missing interactions as opposed to general protein interaction predictions.

Our results showing that Tpk2 functionally affects Sok2 in osmotic stress coupled with previous evidence that the Sok2 sequence contains a consensus PKA phosphorylation site at amino acids 595 to 598 [7], [78] and that PKA phosphorylates Sok2 in other conditions, suggests that the predicted interaction warrants direct experimental validation. Despite their high sequence similarity, the three Tpk's have distinct sets of substrates [82] so confirmatory future work must specifically examine Tpk2 phosphorylation. Because *in vivo* verification of a kinase-substrate interaction is challenging, the next step experimentally will be to show that Tpk2 phosphorylates Sok2 in osmotic stress *in vitro*. Peptide arrays and kinase assays have been used to validate computational phosphorylation predictions *in vitro* [83]. Proteome chips did not detect Sok2 as a Tpk2 substrate *in vitro* [82], highlighting the need for osmotic stress-specific experiments in order to validate our condition-specific prediction. Following *in vitro* confirmation any number of *in vivo* strategies could be used to decisively validate the interaction (see Morandell et al. [84] for a review). For instance, electrophoretic mobility shifts in kinase deletion strains can provide *in vivo* evidence of phosphorylation and validate *in vitro* interactions [82], [83].

Our analysis comparing the set of Sok2 targets and affected *TPK2* knockout (KO) genes with other binding and KO experiments indicated that the overlap between these two sets lies close to the top 10% in both tests. It is not surprising that the deletion of other genes also leads to the differential expression of some Sok2 targets, but the fact that this occurs for only a fraction of experiments suggests that our KO holds against the statistical background. Further, of the 31 KOs with a higher overlap, none correspond to protein products that directly bind to Sok2 according to STRING. As for the overlap between the other TF targets and our *TPK2* KO set, again, it is not surprising that other TFs were affected by the KO because deletions can affect both direct binding partners and proteins further downstream. The more significant Tpk2-TF associations do not correspond to direct binding in the interaction network — the average distance in the interaction network is 4.8 edges — which suggests that these are not candidates for missing interactions.

### Applications to other species and domains

Recently, there has been a great increase in the amount of experimentally derived protein interaction data in several species [85] and in our ability to experimentally query host-environments and host-pathogen interactions [9]. Given these networks, the problem of identifying response pathways can now be tackled in multiple species. A key problem in such studies is dealing with missing interactions, as these prevent algorithms from recovering the correct information flow. The method we presented in this paper is the first to address this issue in a pathway-specific context and can be applied to any species for which such data exists. Further, our method may have use in other domains, for example, in network design where the goal is to reduce routing lags or to aid the flow of information between entities in a network.

## Supporting Information

Figure S1 **The instances of (A) Shortcuts and (B) Shortcuts-X used in the reduction from X3C.**
(TIFF)

Figure S2 **The prediction accuracy of each method using the unoriented STRING PPI network.** (A) Accuracy in identifying STRING potential edges. (B) Accuracy in identifying STRING potential edges that are also HOG-relevant.
(TIFF)

Figure S3 **The prediction accuracy of each method using a hop-restriction length of 4.** (A) Accuracy in identifying STRING potential edges. (B) Accuracy in identifying STRING potential edges that are also HOG-relevant.
(TIFF)

Table S1 **List of affected genes following** ***TPK2*** **knockout.**
(XLS)

Text S1 **Supplementary information.**
(PDF)
