# Supplementary figures and images for: A Network-based Approach for Predicting Missing Pathway Interactions
Source: PLoS Comput Biol. 2012 Aug 16;8(8):e1002640. doi: 10.1371/journal.pcbi.1002640 (PMC3420932; doi:10.1371/journal.pcbi.1002640)

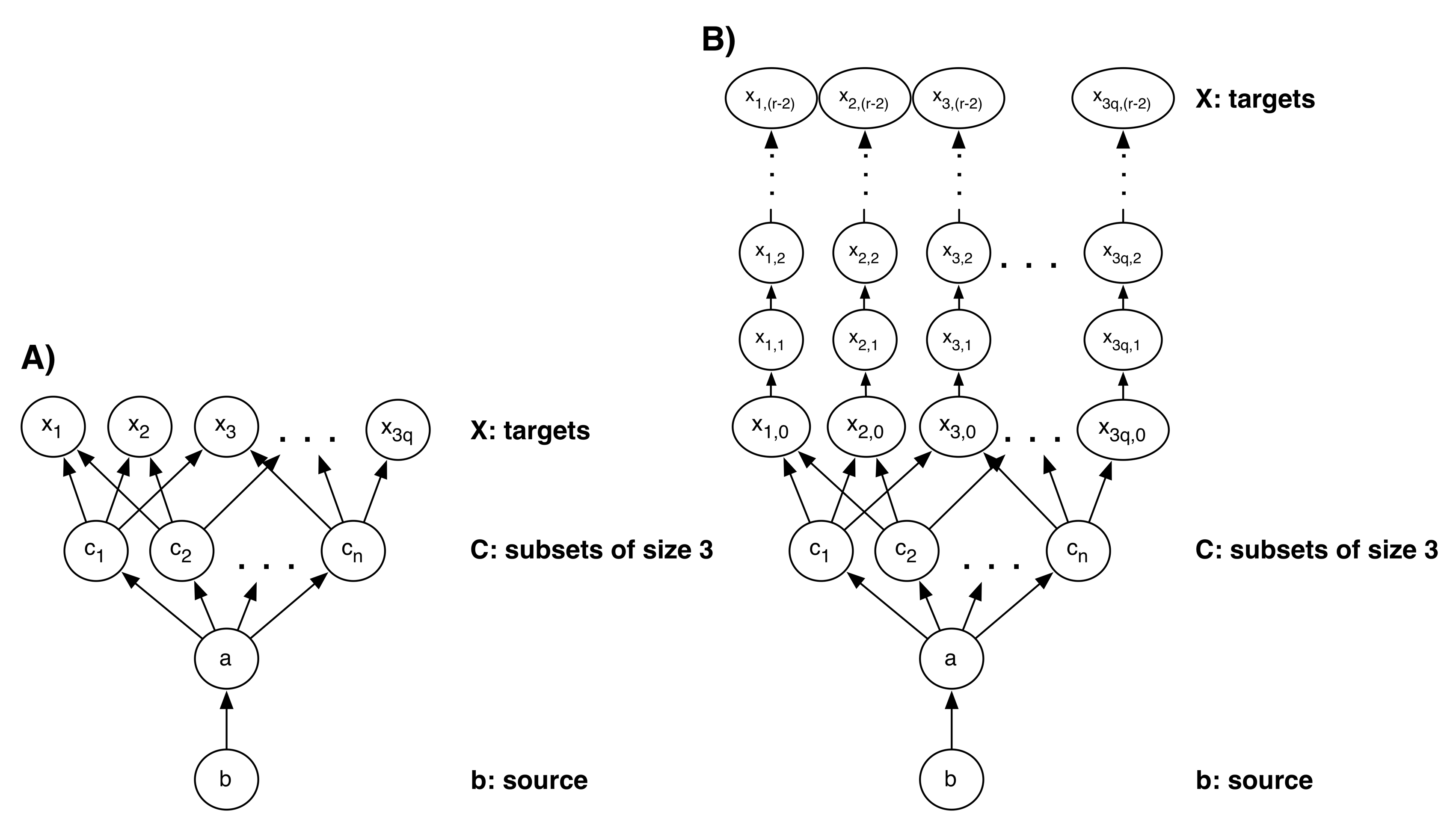

Supplement: Figure S1 — The instances of (A) Shortcuts and (B) Shortcuts-X used in the reduction from X3C. (TIFF) [file pcbi.1002640.s001.tiff]

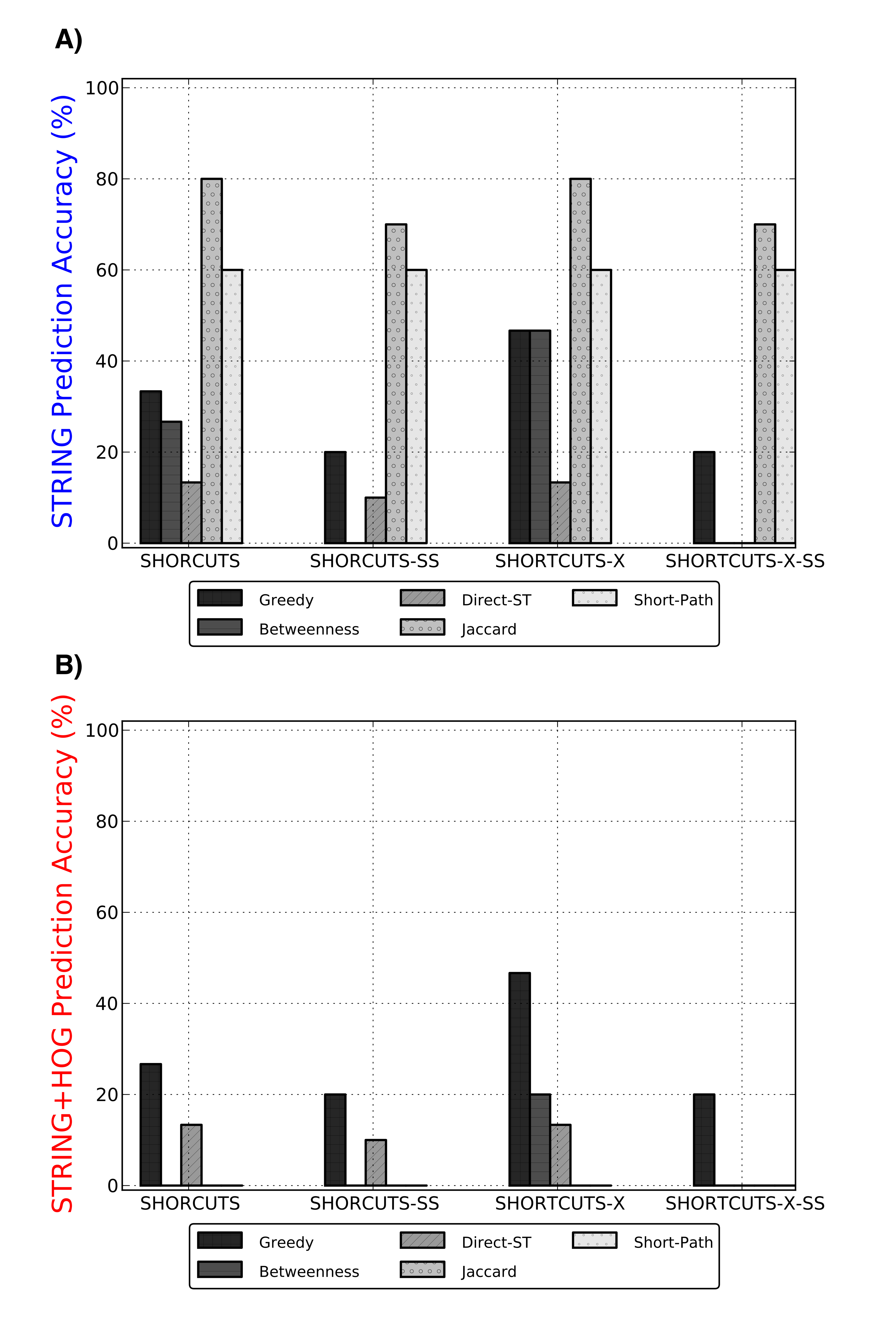

Supplement: Figure S2 — The prediction accuracy of each method using the unoriented STRING PPI network. (A) Accuracy in identifying STRING potential edges. (B) Accuracy in identifying STRING potential edges that are also HOG-relevant. (TIFF) [file pcbi.1002640.s002.tif]

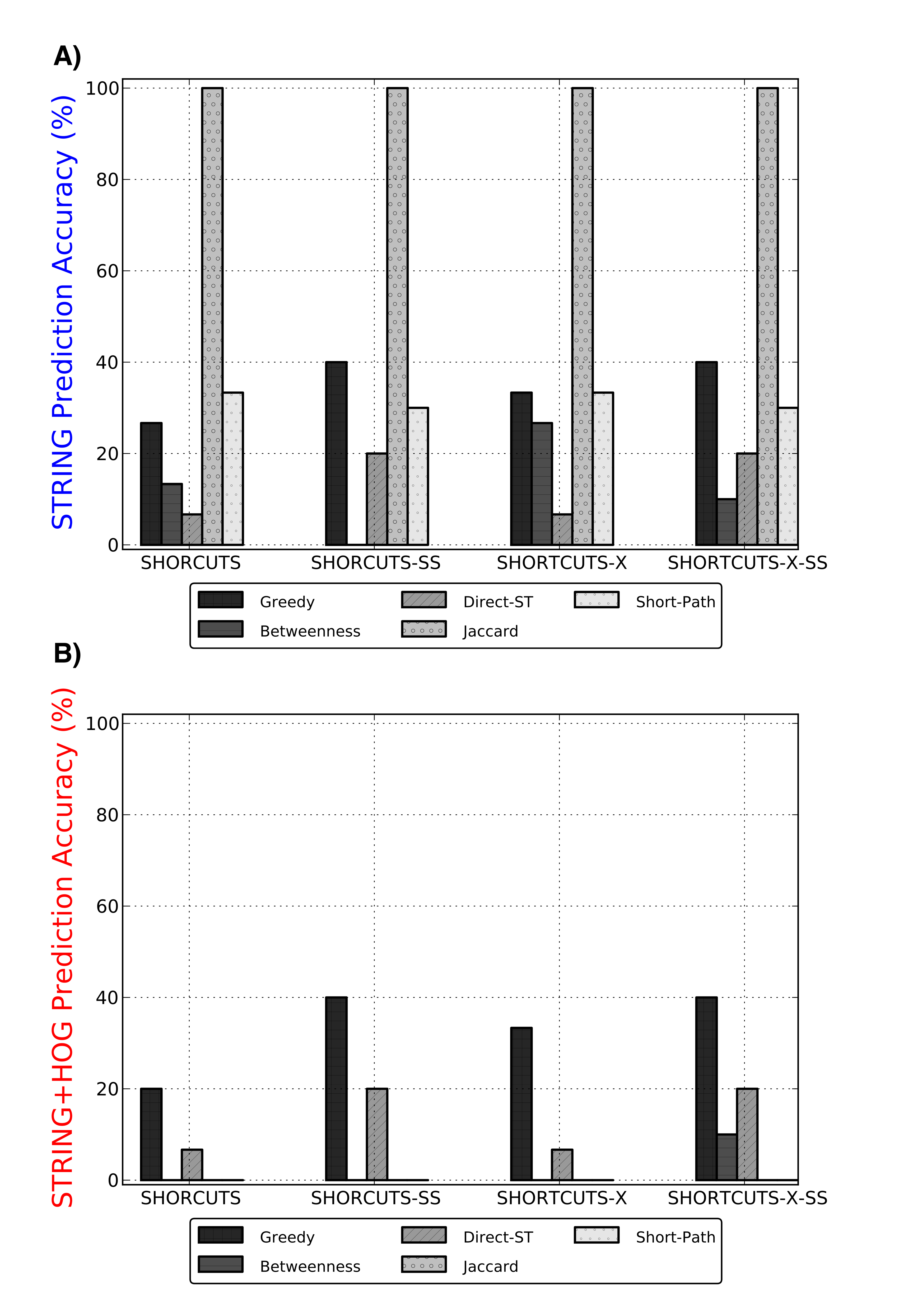

Supplement: Figure S3 — The prediction accuracy of each method using a hop-restriction length of 4. (A) Accuracy in identifying STRING potential edges. (B) Accuracy in identifying STRING potential edges that are also HOG-relevant. (TIFF) [file pcbi.1002640.s003.tiff]
