# Supplementary material for: A Network-based Approach for Predicting Missing Pathway Interactions
Source: PLoS Comput Biol. 2012 Aug 16;8(8):e1002640. doi: 10.1371/journal.pcbi.1002640 (PMC3420932; doi:10.1371/journal.pcbi.1002640)
Supplement: Text S1 — Supplementary information. (PDF) [file pcbi.1002640.s005.pdf]

A Network-based Approach for Predicting Missing Pathway Interactions

Supplementary Materials

Saket Navlakha, Anthony Gitter, Ziv Bar-Joseph
Carnegie Mellon University

1 The edge prediction problems are NP-hard

Theorem 1. *The* SHORTCUTS *problem is NP-hard.*

Proof. Similar to Schoone et al. [1] we reduce from a modified (but equally hard) version of exact cover by three sets (X3C). In X3C, we are given a set of elements $\mathcal{X} = \{x_1, x_2, \ldots, x_{3q}\}$ and a collection $\mathcal{C}$ of 3-element subsets of $\mathcal{X}$. We add the constraint that each element in $\mathcal{X}$ occurs in at least one member of $\mathcal{C}$. We want to find an exact cover of $\mathcal{C}$, i.e. a subset $\mathcal{C}' \subseteq \mathcal{C}$, where $|\mathcal{C}'| = q$, such that every element in $\mathcal{X}$ occurs in exactly one member of $\mathcal{C}'$.

We use the instance of SHORTCUTS shown in Figure S1A. Let the $3q$ pairs between b and all nodes in $\mathcal{X}$ represent the source-target pairs. Below we show that $\mathcal{C}'$ is an exact three-cover of $\mathcal{C}$ if and only if after adding $k = q$ edges to G, the average pairwise distance (APD) between source-target pairs is 2.

X3C $\Rightarrow$ SHORTCUTS: Given a solution $\mathcal{C}'$ to X3C, we add an edge from b to each member in $\mathcal{C}'$ (where $|\mathcal{C}'| = k$). As a result, for every $x \in \mathcal{X}$ there exists a $\mathcal{C}'_x$ that is connected to x. Thus, for every target $x \in \mathcal{X}$ there exists a path from source $b \to \mathcal{C}'_x \to x$ of length exactly 2. Thus, the APD between all source-target pairs is 2.

SHORTCUTS $\Rightarrow$ X3C: We need to show that a solution to SHORTCUTS corresponds to an exact three-covering of $\mathcal{C}$. There are five classes of edges that can be added to G. We consider each case and show how the optimal solution must only consist of edges from b to members of $\mathcal{C}$ and that these edges define a solution to X3C.

Case 1 [edges from $a \to x$]: Any edge added from a to some x_i can be replaced by the corresponding edge from b to x_i without increasing the APD. Thus, in the optimal solution there will be zero edges of this type.

Case 2 [edges from $c \to x$]: We argue that any (c_i, x_j) edge can only decrease the distance from b to x_j and no other $x_k \in \mathcal{X}$. This is because the path from b to x_k that uses (c_i, x_j) must have length ≥ 3. In particular, it takes ≥ 1 hop to go from b to c_i; 1 hop to go from c_i to x_j; and ≥ 1 hop to go from x_j to x_k. However, initially (prior to adding any edges), every x_k belongs to at least one member of $\mathcal{C}$, and so $d(b, x_k) = 3$. Thus, any (c_i, x_j) edge can be replaced by (b, x_j) without increasing the APD.

Case 3 [edges from $x \to x$]: Similarly, any edge from x_i to x_j can only help decrease the distance from b to x_j and no other $x_k \in \mathcal{X}$. Thus, these edges can also be replaced by (b, x_j) without increasing the APD.

Case 4 [backwards edges from $x \to a, x \to c, x \to b, c \to a, c \to b$, or $a \to b$]: Similarly, it is easy to see that these edges cannot be used to decrease the distance between any pair to < 3.

Case 5 [combination of edges from $b \to x$ and $b \to c$]: In the optimal solution, let there be s edges between b and some x's, and $(k - s)$-edges between b and some c's. Below, we argue that $s = 0$.

Each edge from b to x_i yields $d(b, x_i) = 1$. Each of the $(k - s)$ edges from b to c_i yields $d(b, x_j) = 2$ for each x_j linked to from c_i (and where x_j was not linked to directly). All remaining x's have distance 3 from b.

Thus, after $k = q$ edges are added the APD equals:

$$= \frac{1(s) + 2(3(q - s)) + 3(3q - s - 3(q - s))}{3q} \tag{1}$$

$$= \frac{6q + s}{3q}. \tag{2}$$

The first term in Equation (1) corresponds to (b, x_i) edges and contributes distance 1 for each x_i. The second term corresponds to the (b, c_k) edges each of which yields a distance of 2 for up to three x's linked to from c_k. The third term corresponds to the remaining x's whose distance was not reduced and which remains at 3. For the APD to equal 2, it must be that $s = 0$, which implies that there are exactly q edges from b to some c's in the optimal solution.

As a result, for a valid solution to exist, $d(b, x_i) = 2$ for all $x_i \in \mathcal{X}$. This is only possible if there exists a subset $\mathcal{C}' \subset \mathcal{C}$, which contains exactly q elements and such that every $x \in \mathcal{X}$ is linked to from some $c \in \mathcal{C}'$. Because there are $3q$ total x's, it must be that each chosen $c \in \mathcal{C}'$ links to a unique set of x's. Thus, $\mathcal{C}'$ represent an exact cover of $\mathcal{X}$.

Finally, the instance G in Figure S1A can be constructed in polynomial time from an instance of X3C defined by $\mathcal{X}$ and $\mathcal{C}$. Hence, the SHORTCUTS problem is NP-complete.

□

Theorem 2. *The* SHORTCUTS-X *problem is NP-hard for all $r \geq 2$.*

Proof. For brevity, we omit the proof but provide the instance used in the reduction from X3C (Figure S1B). Each x_i from the previous instance is now connected to a $(r-2)$-long string of nodes. This is done essentially as a means to "pump up" the distance between the sources and targets such that each source-target pair will be exactly r hops away after k edges are added. A similar analysis follows. □

Note that because the reduction instance used only one source, the SHORTCUTS-SS and SHORTCUTS-X-SS variants are also both NP-hard.

2 Predictions using the unoriented network

To show that the orientation step is indeed useful in extracting HOG paths given sources and targets, we ran each algorithm on the *unoriented* STRING PPI network (Figure S2). We found that for both hop-restricted objective functions, the Greedy algorithm makes more HOG-relevant predictions when using the oriented network (53% vs. 46% for SHORTCUTS-X and 40% vs. 20% for SHORTCUTS-X-SS, compared to using the unoriented network). Moreover, the global methods (Short-Path and

Jaccard) also benefited significantly from the orientation, which implies that defining network neighbors more precisely can help in identifying putative interactions.

3 Predictions using a hop-restriction length of 4

To explore the sensitivity of our results to the hop-restriction length, we repeated our computational experiments using a hop-restriction length of $r = 4$. Overall, we found similar qualitative performance for the algorithms when predicting from amongst all possible edges (Figure S3). However, when predicting from amongst the potential set, we found only a few overlapping predictions with those made when the hop length was 5. Interestingly, these included the well-known HOG interactions Hog1→Cin5, Hog1→Msn2, and Hog1→Msn4, suggesting that the most confident and likely predictions are not wholly affected by the decreased hop restriction. Of course, some different predictions are also to be expected; for example, using a hop length of 4, the algorithm makes predictions for Sho1→Hog1 and Ste50→Hog1. While these predictions make sense algorithmically, they do not make sense biologically because they attempt to shortcut the sources of the pathway directly to a core node (Hog1). This suggests that 4 hops may be too restrictive and may motivate using a hop restriction of 5 in future efforts.

4 Predictions without using the STRING-derived edge weights

To understand how valuable the STRING-derived edge weights are to our approach, we used the Greedy algorithm to predict edges from the STRING potential set without considering the weight of the edge. (Each potential edge was given a default weight of 0.) In this test, 1 of the top 10 predicted edges overlapped with the predictions when using the STRING-derived weights (P-value = $1.5\mathrm{e}^{-4}$, Fisher's exact test), and this was true for both Shortcuts and Shortcuts-X. When using the weights, 3 of the top predictions for Shortcuts-X connected well-known interactors in the HOG pathway (Hog1→Msn2,Msn4,Cin5) yet none of these predictions emerged in the weight-less predictions. For Shortcuts, several interactions were predicted involving Hot1 (Hot1→Msn2,Msn4,Smp1), all of which have evidence for physical interaction in BioGRID, but none of which were made when using the weights. Thus, as expected, leveraging the weights leads to very different predictions and suggests that there may be multiple ways of connecting HOG sources to targets. Further, this result demonstrates that our objectives are well-defined and are able to uncover some missing edges with or without weights.

5 Microarray analysis and processing

The *tpk2*Δ strain was taken from the Yeast Deletion Library (BY4741 background). Cells were grown to logarithmic phase in SC medium (OD=0.5), washed, and resuspended in SC medium with 1M sorbitol for 30 minutes. The cells were harvested, pelleted, and frozen for further analysis. Total RNA was extracted using MasterPure™ yeast RNA purification kit (Epicentre). The samples were amplified, labeled, hybridized to custom Agilent microarrays (GEO platform GPL14666), and scanned using standard Agilent protocols, reagents, and instruments. Significance analysis of microarrays [2] was run using the two class unpaired response type, 1000 permutations, minimum twofold expression change, the delta that yielded a false discovery rate of at most 0.2, and de-

fault values for all other settings. Genes for which both control measurements or both treatment measurements were missing were discarded.

The custom Agilent microarrays (GEO platform GPL14666) require normalization for a known probe distance bias. Probe distance, the distance between the probe and the 3' UTR end, was derived from reported 3' UTR lengths [3], and we used the average length for genes missing data. Gene expression was normalized by subtracting the Lowess fit of the probe distance and expression and then performing standard quantile normalization. Control probes were discarded, and probe expression was aggregated to the gene level by taking the median. Normalized expression data has been deposited in GEO with accession number GSE28213. Differentially expressed genes were compared to TF binding interactions [4] using the version of the dataset with a 0.005 P-value threshold and no motif conservation requirement.

6 Testing significance versus other perturbation assays

We used the Rosetta compendium[5] of 300 knockout (KO) expression experiments and compared the overlap between differentially expressed (DE) genes in each experiment with the list of Sok2 targets. DE genes in each experiment were identified as those with P-value < 0.005 (the same value was used as the Sok2 TF binding threshold). For each experiment, we computed the significance in overlap of the DE genes and the Sok2 targets using Fishers exact test. Of 301 experiments, only 31 (10.3%) had a lower P-value than the one obtained from our *TPK2* KO.

It is not surprising that the deletion of other genes also leads to the differential expression of some Sok2 targets, but the fact that this occurs for only a fraction of experiments suggests that our KO holds against the statistical background. The physical interaction network provides evidence that, unlike the Tpk2→Sok2 interaction, the other KOs more significantly associated with Sok2 represent indirect relationships. None of the other corresponding protein products directly bind Sok2 according to STRING. Rather, they affect Sok2s bound genes via indirect cascades with an average of 3.5 protein interactions between the KO and Sok2 in the network. This suggests that simply looking at KO significance is not enough and that an approach like ours is necessary to find direct interactions.

In the other direction, we considered 117 additional TFs [4] and measured how many of them were affected by the *TPK2* deletion. For each TF, we computed the overlap between the set of DE genes in our *TPK2* KO and the set of TF targets and computed significance of the overlap using Fishers exact test. Similar as the test above, of the 118 tests only 14 (11.9%) had a lower P-value than our predicted Tpk2-Sok2 pair. Again, it is not surprising that other TFs were affected by the KO because deletions can affect both direct binding partners and proteins further downstream. The more significant Tpk2-TF associations do not correspond to direct binding in the interaction network — the average distance in the interaction network is 4.8 edges — which suggests that these are not candidates for missing interactions.

References

[1] A. A. Schoone, H. L. Bodlaender, and J. Van Leeuwen. Diameter increase caused by edge deletion. *J. Graph Theory*, 11(3):409–427, 1987.

[2] V. G. Tusher, R. Tibshirani, and G. Chu. Significance analysis of microarrays applied to the ionizing radiation response. *Proc. Natl. Acad. Sci. USA*, 98(9):5116–5121, 2001.

[3] U. Nagalakshmi, Z. Wang, K. Waern, C. Shou, D. Raha, M. Gerstein, and M. Snyder. The transcriptional landscape of the yeast genome defined by rna sequencing. *Science*, 320(5881):1344–1349, 2008.

[4] K. D. MacIsaac, T. Wang, D. B. Gordon, D. K. Gifford, G. D. Stormo, and E. Fraenkel. An improved map of conserved regulatory sites for saccharomyces cerevisiae. *BMC Bioinformatics*, 7:113, 2006.

[5] T. R. Hughes, M. J. Marton, A. R. Jones, C. J. Roberts, R. Stoughton, C. D. Armour, H. A. Bennett, E. Coffey, H. Dai, Y. D. He, M. J. Kidd, A. M. King, M. R. Meyer, D. Slade, P. Y. Lum, S. B. Stepaniants, D. D. Shoemaker, D. Gachotte, K. Chakraburtty, J. Simon, M. Bard, and S. H. Friend. Functional discovery via a compendium of expression profiles. *Cell*, 102(1):109–126, Jul 2000.
